# Hyperphagia and Obesity in Prader–Willi Syndrome: *PCSK1* Deficiency and Beyond?

**DOI:** 10.3390/genes9060288

**Published:** 2018-06-07

**Authors:** Bruno Ramos-Molina, María Molina-Vega, José C. Fernández-García, John W. Creemers

**Affiliations:** 1 Laboratory of Cellular and Molecular Endocrinology, Institute of Biomedical Research in Malaga (IBIMA), Virgen de la Victoria University Hospital, 29010 Malaga, Spain; brunoramosmolina@gmail.com; 2 Department of Endocrinology, Virgen de la Victoria University Hospital, 29010 Malaga, Spain; molinavegamaria@gmail.com; 3 CIBER Fisiopatología de la Obesidad y Nutrición (CIBERObn CB06/003), Instituto de Salud Carlos III, 28029 Madrid, Spain; 4 Laboratory of Biochemical Neuroendocrinology, Department of Human Genetics, KU Leuven, B-3000 Leuven, Belgium; john.creemers@kuleuven.be

**Keywords:** Prader–Willi syndrome, *PCSK1* deficiency, obesity, hyperphagia, hypothalamus

## Abstract

Prader–Willi syndrome (PWS) is a complex genetic disorder that, besides cognitive impairments, is characterized by hyperphagia, obesity, hypogonadism, and growth impairment. Proprotein convertase subtilisin/kexin type 1 (*PCSK1*) deficiency, a rare recessive congenital disorder, partially overlaps phenotypically with PWS, but both genetic disorders show clear dissimilarities as well. The recent observation that *PCSK1* is downregulated in a model of human PWS suggests that overlapping pathways are affected. In this review we will not only discuss the mechanisms by which PWS and *PCSK1* deficiency could lead to hyperphagia but also the therapeutic interventions to treat obesity in both genetic disorders.

## 1. Genetic Disorders Related to Hyperphagia and Obesity

Obesity, defined as a disproportionate body weight for height with an excessive accumulation of adipose tissue [1], is the result of a net imbalance of caloric intake over energy expenditure over time [2], caused by several genetic and nongenetic risk factors [3]. Genetic forms of obesity have been historically subdivided into (1) Mendelian (monogenic) nonsyndromic obesity, (2) Mendelian syndromic obesity, and (3) polygenic obesity [4].

Polygenic obesity is the most common form of obesity and is caused by the presence of multiple DNA variations in multiple genes that have a small effect on body weight regulation [5]. It is estimated that more than 200 low-risk common genetic variants exist. Many risk prediction models have been developed in an attempt to determine the genetic susceptibility which, combined with an unhealthy lifestyle, leads to obesity [6].

Monogenic forms of obesity are rare and are usually characterized by early-onset obesity associated with endocrine disorders, mainly due to mutations in genes of the leptin/melanocortin axis involved in food intake regulation [7]. Mutations in several genes implicated in the leptin–melanocortin pathway have been shown to lead to autosomal recessive forms of obesity such as leptin (*LEP*), leptin receptor (*LEPR*), melanocortin 4 receptor (*MC4R*), pro-opiomelanocortin (*POMC*), prohormone convertase subtilisin/kexin type 1 (*PCSK1*), single-minded 1 (*SIM1*), neurotrophic tyrosine kinase receptor type 2 (*NTRK2*), dedicator of cytokinesis 5 (*DOCK5*), kinase suppressor of Ras2 (*KSR2*), or tubby-like protein (*TUB*) [7]. Leptin deficiency was the first cause of monogenic obesity to be demonstrated in a human patient [8]. Variants in the *MC4R* gene represent the most common cause of monogenic obesity [9].

Syndromic obesity can be defined as the presence of obesity along with additional characteristics, including intellectual disability, dysmorphic features, and congenital abnormalities affecting specific organ systems [6]. Many such obesity syndromes have been described in the literature thus far, as compiled by Kaur et al. [6], and Prader–Willi syndrome is one of the most prevalent.

Prader–Willi syndrome (PWS) is a multisystemic complex genetic disorder resulting from the absence of expression of the paternally inherited genes on chromosome 15q11.2-q13 (alleles from the maternally contributed chromosome are normally inactivated by epigenetic factors and not expressed) [10]. This lack of expression is due, in 65–75% of cases, to paternal deletion [10]. In about 25% of cases, maternal disomy 15 is found [11]. The remaining individuals have an imprinting defect of this region (<3%) and chromosomal translocations or rearrangements of the 15q11-q13 region have been reported too on very rare occasions [12]. PWS has an estimated prevalence of 1 in 10,000 to 30,000, with equal numbers of male and females affected [13]. The clinical manifestations of PWS are severe neonatal hypotonia, intellectual disabilities and behavioral problems [12], short stature and growth hormone deficiency, dysmorphic features, and hypogonadism [10]. Traditionally, two nutritional phases have been described in PWS: the first one characterized by failure to thrive in the context of poor feeding and hypotonia and the second one characterized by hyperphagia which leads to obesity. However, Miller et al. [14] have differentiated seven phases, denoting a higher complexity. Other endocrine issues such as central adrenal insufficiency, hypothyroidism, impaired glucose tolerance, and diabetes mellitus can also be found in PWS in addition to other findings including sleep abnormalities, strabismus, hip dysplasia, scoliosis, recurrent respiratory infections, and others (bone fractures caused by osteopenia, leg edema and ulceration, skin picking, altered temperature sensation, decreased saliva flow, high vomiting threshold, or seizures) [10].

## 2. Prader–Willi Syndrome and Obesity: Does *PCSK1* Activity Matter?

Individuals with inactivating mutations in the *PCSK1* gene have several phenotypic similarities with PWS patients, which raises the possibility of a role of PC1/3 (the gene product of *PCSK1*) activity in several clinical aspects of PWS patients, including severe early-onset obesity, hypogonadotropic hypogonadism, and growth retardation [15]. One of the most evident phenotype characteristics in both PWS and PC1/3-deficient subjects is hyperphagic obesity. The fact that induced pluripotent stem cell (iPSC)-derived neurons from PWS patients display reduced levels of PC1/3 supports the idea that hyperphagia can be caused by impairment in central PC1/3 activity [16]. In the central nervous system (CNS), the hypothalamus is a specific region that includes different neuronal populations involved in feeding behavior and energy homeostasis. In the hypothalamus, PC1/3 expression is particularly high in both proopiomelanocortin (POMC) neurons and agouti-related peptide/neuropeptide Y (AgRP/NPY) neurons, two leptin-responsive neuronal populations within the arcuate nucleus (ARC). PC1/3 activity is essential for both POMC and proAGRP processing in the ARC [17]. PC1/3 proteolytically processes POMC to adrenocorticotropic hormone (ACTH), which is further cleaved into α-melanocyte-stimulating hormone (α-MSH) by PC2. Since PC2 itself is not capable of producing ACTH from POMC in vitro, one would expect the absence of PC1/3 activity to result in the total loss of α-MSH, a neuropeptide with potent anorexigenic actions, by binding the melanocortin receptor 4 (MC4R). However, a recent study reported that PC1/3 deficiency in human embryonic stem cell (hESC)-derived hypothalamic neurons increases the levels of unprocessed POMC and the expression of melanocortin receptors, and reduces ACTH secretion; but, conversely, it does not result in a clear reduction of α-MSH levels [18], which is consistent with the fact that the first described *PCSK1* null patients exhibited extreme hyperphagia in early childhood, but this hyperphagia became less outspoken in older patients [19,20]. It should be mentioned that despite the fact that PC1/3 is the main enzyme that converts POMC into ACTH, PC2 or, more likely, other members of the PC family are involved in the POMC-to-ACTH processing in absence of PC1/3. In this regard, in some PC1/3-deficient patients the plasma levels of ACTH were near-normal [19,20]. Besides this, redundancy has been described for PCs in mouse models or cells in which a specific PC has been specifically ablated [21].

On the other hand, AgRP, which is mainly produced by PC1/3 from its precursor proAgRP, is an orexigenic hormone that antagonizes the effects of POMC-derived neuropeptides by direct competition for binding to the MCR4 receptors. Despite the fact that the PC1/3-cleaved form binds more potently to MCR4 than the full-length form, the uncleaved form still has considerable antagonistic activity toward α-MSH at the MC4R [22]. Importantly, hypothalamic proAgRP levels are increased in *PCSK1* knockout mice, suggesting that increased proAgRP could participate in the induction of hyperphagia [22]. In addition, hypothalamic gene expression levels of AgRP are increased in the PWS-like Snord116p-/m+ mice, which may support this idea [16]. 

In addition, recent work has established a new paradigm in the field of neural control of feeding behavior, as it was convincingly demonstrated that AgRP/NPY neurons are the crucial hypothalamic neurons involved in the central response to leptin [23]. Given the clear effect of PC1/3 activity on AgRP maturation AgRP/NPY neurons could be an interesting therapeutic target to treat hyperphagia in both PC1/3-deficient and PWS subjects.

**Figure 1:**
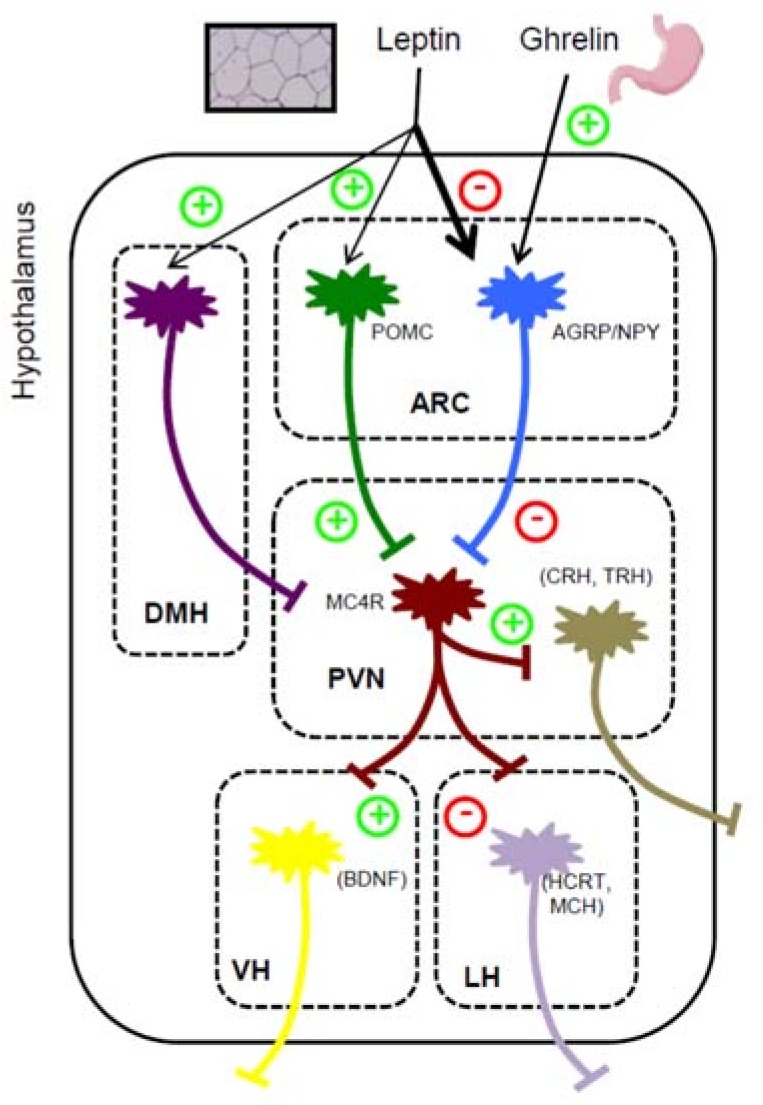
PC1/3-mediated processing of neuropeptides implicated in central regulation of appetite. This figure includes the most important hypothalamic nuclei implicated in feeding behavior. All peptides listed are either confirmed or potential PC1/3 substrates. Abbreviations: AGRP, agouti-related peptide; ARC, arcuate nucleus; BDNF, brain-derived neurotrophic factor; CRH, corticotropin-releasing hormone; DMH, dorsomedial hypothalamus; HCRT, orexin precursor; LH, lateral hypothalamus; MCH, melanin-concentrating hormone; NPY, neuropeptide Y; POMC, pro-opiomelanocortin; PVN, paraventricular nucleus; TRH, thyrotropin-releasing hormone; VH, ventromedial hypothalamus.

Besides the ARC, PC1/3 is present in several other hypothalamic regions including the paraventricular nucleus (PVN), the ventromedial hypothalamus (VMH), and the lateral hypothalamus (LH), where it could be processing other neuropeptides involved in feeding behavior including brain-derived neurotrophic factor (BDNF), corticotropin-releasing hormone (CRH), thyrotropin-releasing hormone (TRH), orexins, and melanin-concentrating hormone (MCH). Among these potential PC1/3 substrates, only TRH is a confirmed product of PC1/3 activity in the hypothalamus [15,24]. BDNF is coexpressed with PC1/3 in certain hypothalamic areas, although there is no direct evidence of PC1/3-mediated proBDNF cleavage so far. In fact, recent work has demonstrated that the loss of PC7, but not PC1/3, in mice reduces proBDNF processing in the hypothalamus [25], indicating that PC1/3 is probably not the major proteolytic enzyme implicated in BDNF maturation. It is worth noting that, taking into consideration the possible impairment of the leptin–melanocortin pathway in both *PCSK1* null and PWS patients, together with the fact that all these neuropeptides are secreted after MC4R activation (Figure 1), their involvement in the induction of hyperphagia could be minimal.

In addition to the CNS, PC1/3 is the enzyme responsible for the activation of several peripheral hormones that are known to influence appetite regulation such as ghrelin, insulin, or glucagon-like peptides. Ghrelin, which is produced from proghrelin by PC1/3 [26], is a gastrointestinal hormone with potent orexigenic properties that is secreted under fasting conditions. In the hypothalamus, ghrelin activates AgRP/NPY neurons, inducing food intake. Remarkably, it is well documented that PWS subjects usually exhibit high levels of circulating ghrelin. However, to the best of our knowledge, circulating ghrelin levels have not been measured in any individual with PC1/3 deficiency. In mice, both Snord116p-/m+ and *PCSK1* null animals display increased transcript levels of preproghrelin in the stomach associated with impaired stomach proghrelin processing [16,26]. 

Insulin is an anabolic hormone produced and secreted by the pancreatic β cells whose primary function is promoting glucose uptake and glycogen synthesis, and inhibiting lipolysis. In the CNS, insulin signaling has been implicated in the control of feeding, hepatic glucose production, and energy homeostasis [27]. In the pancreatic β cell, proinsulin is synthesized in the endoplasmic reticulum and activation of this precursor to insulin requires the sequential action of PC1/3 and PC2, a process that starts in the trans-Golgi and continues in the secretory granules. Consistently, mice lacking PC1/3 display impaired proinsulin processing and increased proinsulin-to-insulin ratios [28,29]. In PC1/3-deficient patients, the levels of proinsulin and 64,65-des-split proinsulin are abnormally high, whereas in control samples this cleavage product is almost undetectable [15,20,30]. In addition, an elevated ratio of proinsulin to insulin during fasting was detected in the plasma of PWS patients and Snord116p-/m+ mice compared with their respective controls, suggesting that proinsulin processing is impaired in PWS as well [16].

## 3. Prader–Willi Syndrome and *PCSK1* Deficiency beyond Obesity: Similarities and Differences

In addition to early-onset obesity and hyperphagia, subjects with PWS and those with *PCSK1* deficiency have other common clinical characteristics. All patients with PWS have some degree of hypogonadism, either primary or central hypogonadism, and in some cases a combination of both [12,13]. Hypogonadism manifests as genital hypoplasia (66–100% of male newborns present cryptorchidism and 76% of female newborns present hypoplasia of the external genitalia [13]), incomplete pubertal development, and infertility in the majority of patients [10]. However, a subset of PWS females may have preservation of fertility [13] and to date four females with PWS have reportedly become pregnant [12]. On the other hand, no cases of paternity in PWS males are yet known [13]. Regarding *PCSK1* deficiency, hypogonadotropic hypogonadism has been reported in 7/21 cases of *PCSK1* deficiency, but in 9/21 cases hypogonadism was absent and in 5/21 it was not reported [15]. Remarkably, the first case of *PCSK1* deficiency reported in the literature, a woman with fertility problems among other endocrinopathies, successfully became pregnant after gonadotropin treatment [19,30], demonstrating that hypogonadism could be reversed by hormonal therapy. 

Short stature is a hallmark of individuals with PWS, and growth hormone (GH) deficiency is the most commonly reported endocrinopathy in these subjects [13]. GH deficiency, leading to reduced growth in patients with *PCSK1* deficiency, has been diagnosed in 5/21 patients, was absent in 9/21 patients, and not reported in 7/21 patients [15]. GH deficiency is likely due to impaired pro-GH-releasing hormone (proGHRH) processing, as *PCSK1* null mice exhibit a defect in the processing of proGHRH to GHRH [28].

Regarding central hypothyroidism, the prevalence in subjects with PWS cannot be clearly established and has been reported as 2.1% to 72.2% [12]; on the other hand, more than half of the individuals with *PCSK1* deficiency exhibit central hypothyroidism, similar to those presenting hypocortisolism [15]. Although, due to the hypothalamic dysfunction present in PWS, these patients are at risk for central adrenal insufficiency, the true prevalence in this population remains unclear, with reported prevalence ranging from 0% to 60% [13].

With respect to glucose metabolism, impaired glucose tolerance is present in many individuals with PWS, presenting as non-insulin-dependent diabetes mellitus in up to 25% of them in adulthood, especially those with significant obesity [10]. In more than 70% of patients with *PCSK1* deficiency, abnormal glucose homeostasis with high proinsulin-to-insulin ratio is found. In several individuals with *PCSK1* deficiency, postprandial hypoglycemia is reported, due to the longer half-life of proinsulin compared to insulin [15].

However, there are also some specific differences between PWS and *PCSK1* deficiency. Almost without exception, individuals with PWS present hypotonia at birth and during the neonatal period [12], improving over time, although adults remain mildly hypotonic [10]. Hypotonia is such a hallmark symptom that PWS needs to be excluded in all cases of unexplained neonatal hypotonia [12]. In addition, PWS patients usually have mild intellectual disability (average intelligence quotient 65) [12]. None of these features have been associated with *PCSK1* deficiency. On the other hand, most subjects with *PCSK1* deficiency have been reported to have diarrhea with malabsorption of fatty acids, amino acids, and monosaccharides very soon after birth [31], usually requiring hospitalization and in several cases resulting in death in early childhood. Interestingly, no clear abnormalities are found in intestinal biopsies and gastrointestinal problems diminish with time, in parallel with the progressive weight gain [15].

## 4. Therapeutic Options for Obesity in PWS and *PCSK1* Patients

Undoubtedly, the management of obesity is one of the most important (and challenging) therapeutic objectives in those patients with PWS and *PCSK1* deficiency. On this point, it is important to note that clinical experience in the management of obesity in subjects with PWS is more profuse than in patients with *PCSK1* deficiency, given the low number of patients with the latter. 

Generally speaking, all patients with PWS and *PCSK1* deficiency, given the early onset of severe obesity, should receive nutritional and exercise advice to prevent excessive weight gain and the development of metabolic derangements, such as hypertension, type 2 diabetes, or dyslipidemia. Specifically, dietary advice should begin in early infancy to prevent inappropriate weight gain [32]. Daily intakes of 600–800 kcal for young children with PWS and 800–1300 kcal for older children and adults with PWS have been recommended to avoid excessive weight gain [33]. Regarding the optimal macronutrient composition of the diet for individuals with PWS or *PCSK1* deficiency, no consensus has yet been reached; some authors suggest a PWS diet consisting of approximately 25% protein, 50% carbohydrate, and 25% fat, whilst others have suggested the use of ketogenic diets or hypocaloric protein-sparing diets [33]. 

Given the increased appetite in both PWS and *PCSK1* deficiency, management of hyperphagia is a priority in these patients. Over the years, several successful strategies have been developed for PWS patients, all aimed at reducing the caloric intake by means of close supervision, offering healthy alternatives, reducing portions, eliminating temptations, setting good examples, and instructing people close to the patients including educational centers [34]. 

Exercise is also a major factor in weight maintenance, and early establishment of a routine of regular daily physical activity should be strongly recommended in these patients. In accordance with general guidelines on physical activity, patients should be advised to engage in at least 150 min of moderate-intensity activity each week, and also to include muscle-strengthening activities that involve all major muscle groups on two or more days a week, tailoring the physical activity to the patient’s physical form and clinical characteristics [32,35].

Although diverse anti-obesity drugs are currently approved for the treatment of obesity (orlistat, naltrexone/bupropion, and liraglutide 3.0 mg), none has been specifically studied in patients with PWS or *PCSK1* deficiency. However, there are some reports indicating that exenatide, a glucagon-like peptide-1 receptor agonist, decreases appetite without change in weight or body mass index (BMI) in the short term (6 months) [36] in patients with PWS. Also, diverse clinical trials are currently evaluating the efficacy of several drugs and interventions in hyperphagia and weight loss in PWS: liraglutide, oxytocin, and tesofensine/metoprolol, amongst others (Table 1).

An interesting approach for weight loss and hyperphagia is the use of drugs that target the endocannabinoid system [37]. In particular, the endocannabinoid/CB1 receptor system seems to be dysregulated in PWS patients and the use of cannabinoid antagonists has been proposed as a therapeutic option for PWS [38]. In fact, a phase II clinical trial is currently testing the effects of cannabidiol in the hyperphagic phenotype in children with PWS (see Table 1). Rimonabant, a CB1 receptor antagonist, was a promising drug in clinical trials for weight loss and hyperphagia, but is no longer available for clinical use due to neuropsychiatric side effects. Interestingly, in rodents, the administration of CB1 antagonists induces hypothalamic PC1/3 protein expression levels but does not affect the synthesis and secretion of α-MSH by the hypothalamus [39].

Finally, despite the fact that bariatric surgery is considered the most effective long-term treatment for severe obesity, published experience in PWS patients has demonstrated poor weight loss efficacy over time and numerous surgical complications [40]. However, a recent report on PWS children and adolescents indicates that laparoscopic sleeve gastrectomy (a type of bariatric surgery) is effective in achieving weight loss and resolution of co-morbidities, without mortality, significant morbidity, or slowing of growth [41]. Also, bariatric surgery has been reported to be effective in reducing body weight and reversing diabetes mellitus in the index patient with *PCSK1* deficiency [30]. Therefore, bariatric surgery can be considered when treating severe obesity in patients with PWS and *PCSK1* deficiency.

## 5. Concluding Remarks

The recent suggestion that some of the major neuroendocrine features of PWS are due to PC1/3 deficiency [16] has triggered a renewed analysis of the similarities and differences in clinical manifestations of both syndromes. A follow-up study by Polex-Wolf and colleagues [42], showing that selective disruption of Snord116 in the mediobasal hypothalami of mice recapitulates the hyperphagia in PWS without affecting the expression of *PCSK1*, challenged this model. Additional studies using cellular models like iPSC-derived neurons from patients and cell-type-specific transgenic mouse models are needed to dissect the role of *PCSK1* in PWS and unveil the molecular mechanisms responsible for both the similarities and differences between the two syndromes. Identification of PC1/3 substrates relevant for hyperphagia and obesity may result in the identification of novel therapeutic targets downstream of this substrate, which might benefit both PWS and PC1/3-deficient patients. Conversely, ongoing clinical trials for the treatment of PWS patients might provide therapeutic benefits for *PCSK1* null patients. Finally, although appealing, these new approaches for the treatment of hyperphagia and obesity in PWS and *PCSK1* deficiency will have to prove their therapeutic potential in general obesity.

## Figures and Tables

**Table 1:** Clinical trials currently recruiting patients or active, conducted in patients with Prader–Willi syndrome (PWS), posted on clinicaltrials.gov (last verified May 2018).

Drug/Device	Phase	Primary Outcome	ClinicalTrials.gov Identifier
Liraglutide	Phase III	Change in body mass index	NCT02527200
Oxytocin	Phase II	Change in hyperphagia	NCT03197662
Oxytocin	Phase II	Change in hyperphagia	NCT02629991
Oxytocin	Phase II/Phase III	Change in eating behavior	NCT02804373
Deep Brain Stimulation	Phase I	Change in waist circumference; mid-upper arm circumference, resting energy expenditure, and body mass index	NCT02297022
Tesofensine/Metoprolol	Phase II	Change in body weight	NCT03149445
Diazoxide Choline	Phase III	Change in hyperphagia	NCT03440814
Cannabidiol	Phase II	Change in hyperphagia	NCT02844933
GLWL-01	Phase II	Change in hyperphagia	NCT03274856
RM-493	Phase II	Change in weight loss and hyperphagia	NCT02311673
Noninvasive brain stimulation	Not Applicable	Change in food craving	NCT02758262

## References

[1] Gonzalez-Muniesa P., Martinez-Gonzalez M.A., Hu F.B., Despres J.P., Matsuzawa Y., Loos R.J.F., Moreno L.A., Bray G.A., Martinez J.A. (2017). Obesity. Nat. Rev. Dis. Primers.

[2] Chung W.K. (2012). An overview of monogenic and syndromic obesities in humans. Pediatr. Blood Cancer.

[3] Hinney A., Vogel C.I., Hebebrand J. (2010). From monogenic to polygenic obesity: Recent advances. Eur. Child Adolesc. Psychiatry.

[4] Loos R.J.F., Janssens A. (2017). Predicting polygenic obesity using genetic information. Cell Metab..

[5] Walley A.J., Asher J.E., Froguel P. (2009). The genetic contribution to non-syndromic human obesity. Nat. Rev. Genet..

[6] Kaur Y., de Souza R.J., Gibson W.T., Meyre D. (2017). A systematic review of genetic syndromes with obesity. Obes. Rev..

[7] Huvenne H., Dubern B. (2014). Monogenic Forms of Obesity.

[8] Montague C.T., Farooqi I.S., Whitehead J.P., Soos M.A., Rau H., Wareham N.J., Sewter C.P., Digby J.E., Mohammed S.N., Hurst J.A. (1997). Congenital leptin deficiency is associated with severe early-onset obesity in humans. Nature.

[9] Vollbach H., Brandt S., Lahr G., Denzer C., von Schnurbein J., Debatin K.M., Wabitsch M. (2017). Prevalence and phenotypic characterization of *MC4R* variants in a large pediatric cohort. Int. J. Obes..

[10] Cassidy S.B., Schwartz S., Miller J.L., Driscoll D.J. (2012). Prader-Willi syndrome. Genet. Med..

[11] Nicholls R.D., Knoll J.H., Butler M.G., Karam S., Lalande M. (1989). Genetic imprinting suggested by maternal heterodisomy in nondeletion Prader-Willi syndrome. Nature.

[12] Angulo M.A., Butler M.G., Cataletto M.E. (2015). Prader-Willi syndrome: A review of clinical, genetic, and endocrine findings. J. Endocrinol. Investig..

[13] Heksch R., Kamboj M., Anglin K., Obrynba K. (2017). Review of Prader-Willi syndrome: The endocrine approach. Transl. Pediatr..

[14] Miller J.L., Lynn C.H., Driscoll D.C., Goldstone A.P., Gold J.A., Kimonis V., Dykens E., Butler M.G., Shuster J.J., Driscoll D.J. (2011). Nutritional phases in Prader-Willi syndrome. Am. J. Med. Genet. A.

[15] Stijnen P., Ramos-Molina B., O’Rahilly S., Creemers J.W. (2016). PCSK1 mutations and human endocrinopathies: from obesity to gastrointestinal disorders. Endocr. Rev..

[16] Burnett L.C., LeDuc C.A., Sulsona C.R., Paull D., Rausch R., Eddiry S., Carli J.F., Morabito M.V., Skowronski A.A., Hubner G. (2017). Deficiency in prohormone convertase PC1 impairs prohormone processing in Prader-Willi syndrome. J. Clin. Investig..

[17] Ramos-Molina B., Martin M.G., Lindberg I. (2016). *PCSK1* Variants and Human Obesity. Prog. Mol. Biol. Transl. Sci..

[18] Wang L., Sui L., Panigrahi S.K., Meece K., Xin Y., Kim J., Gromada J., Doege C.A., Wardlaw S.L., Egli D. (2017). PC1/3 deficiency impacts pro-opiomelanocortin processing in human embryonic stem cell-derived hypothalamic neurons. Stem Cell Rep..

[19] Jackson R.S., Creemers J.W., Ohagi S., Raffin-Sanson M.L., Sanders L., Montague C.T., Hutton J.C., O’Rahilly S. (1997). Obesity and impaired prohormone processing associated with mutations in the human prohormone convertase 1 gene. Nat. Genet..

[20] Jackson R.S., Creemers J.W., Farooqi I.S., Raffin-Sanson M.L., Varro A., Dockray G.J., Holst J.J., Brubaker P.L., Corvol P., Polonsky K.S. (2003). Small-intestinal dysfunction accompanies the complex endocrinopathy of human proprotein convertase 1 deficiency. J. Clin. Investig..

[21] Creemers J.W., Khatib A.M. (2008). Knock-out mouse models of proprotein convertases: Unique functions or redundancy?. Front. Biosci..

[22] Creemers J.W., Pritchard L.E., Gyte A., Le Rouzic P., Meulemans S., Wardlaw S.L., Zhu X., Steiner D.F., Davies N., Armstrong D. (2006). Agouti-related protein is posttranslationally cleaved by proprotein convertase 1 to generate agouti-related protein (AGRP)83-132: Interaction between AGRP83-132 and melanocortin receptors cannot be influenced by syndecan-3. Endocrinology.

[23] Xu J., Bartolome C.L., Low C.S., Yi X., Chien C.H., Wang P., Kong D. (2018). Genetic identification of leptin neural circuits in energy and glucose homeostases. Nature.

[24] Sanchez V.C., Goldstein J., Stuart R.C., Hovanesian V., Huo L., Munzberg H., Friedman T.C., Bjorbaek C., Nillni E.A. (2004). Regulation of hypothalamic prohormone convertases 1 and 2 and effects on processing of prothyrotropin-releasing hormone. J. Clin. Investig..

[25] Wetsel W.C., Rodriguiz R.M., Guillemot J., Rousselet E., Essalmani R., Kim I.H., Bryant J.C., Marcinkiewicz J., Desjardins R., Day R. (2013). Disruption of the expression of the proprotein convertase PC7 reduces BDNF production and affects learning and memory in mice. Proc. Natl. Acad. Sci. USA.

[26] Zhu X., Cao Y., Voogd K., Steiner D.F. (2006). On the processing of proghrelin to ghrelin. J. Biol. Chem..

[27] Porte D., Baskin D.G., Schwartz M.W. (2005). Insulin signaling in the central nervous system: A critical role in metabolic homeostasis and disease from *C. elegans* to humans. Diabetes.

[28] Zhu X., Zhou A., Dey A., Norrbom C., Carroll R., Zhang C., Laurent V., Lindberg I., Ugleholdt R., Holst J.J. (2002). Disruption of PC1/3 expression in mice causes dwarfism and multiple neuroendocrine peptide processing defects. Proc. Natl. Acad. Sci. USA.

[29] Zhu X., Orci L., Carroll R., Norrbom C., Ravazzola M., Steiner D.F. (2002). Severe block in processing of proinsulin to insulin accompanied by elevation of des-64,65 proinsulin intermediates in islets of mice lacking prohormone convertase 1/3. Proc. Natl. Acad. Sci. USA.

[30] O’Rahilly S., Gray H., Humphreys P.J., Krook A., Polonsky K.S., White A., Gibson S., Taylor K., Carr C. (1995). Brief report: Impaired processing of prohormones associated with abnormalities of glucose homeostasis and adrenal function. N. Engl. J. Med..

[31] Martin M.G., Lindberg I., Solorzano-Vargas R.S., Wang J., Avitzur Y., Bandsma R., Sokollik C., Lawrence S., Pickett L.A., Chen Z. (2013). Congenital proprotein convertase 1/3 deficiency causes malabsorptive diarrhea and other endocrinopathies in a pediatric cohort. Gastroenterology.

[32] Cassidy S.B., Driscoll D.J. (2009). Prader-Willi syndrome. Eur. J. Hum. Genet..

[33] Irizarry K.A., Miller M., Freemark M., Haqq A.M. (2016). Prader Willi Syndrome: Genetics, Metabolomics, Hormonal Function, and New Approaches to Therapy. Adv. Pediatr..

[34] Butler M.G. (2006). Management of obesity in Prader-Willi syndrome. Nat. Clin. Pract. Endocrinol. Metab..

[35] Garvey W.T., Mechanick J.I., Brett E.M., Garber A.J., Hurley D.L., Jastreboff A.M., Nadolsky K., Pessah-Pollack R., Plodkowski R. (2016). Reviewers of the AACE/ACE Obesity Clinical Practice Guidelines. American Association of Clinical Endocrinologists and American College of Endocrinology Comprehensive Clinical Practice Guidelines for Medical Care of Patients with Obesity. Endocr. Pract..

[36] Salehi P., Hsu I., Azen C.G., Mittelman S.D., Geffner M.E., Jeandron D. (2017). Effects of exenatide on weight and appetite in overweight adolescents and young adults with Prader-Willi syndrome. Pediatr. Obes..

[37] Koch M. (2017). Cannabinoid receptor signaling in central regulation of feeding behavior: A Mini-Review. Front. Neurosci..

[38] Knani I., Earley B.J., Udi S., Nemirovski A., Hadar R., Gammal A., Cinar R., Hirsch H.J., Pollak Y., Gross I. (2016). Targeting the endocannabinoid/CB1 receptor system for treating obesity in Prader-Willi syndrome. Mol. Metab..

[39] Koch M., Varela L., Kim J.G., Kim J.D., Hernandez-Nuno F., Simonds S.E., Castorena C.M., Vianna C.R., Elmquist J.K., Morozov Y.M. (2015). Hypothalamic POMC neurons promote cannabinoid-induced feeding. Nature.

[40] Alqahtani A.R., Elahmedi M.O., Al Qahtani A.R., Lee J., Butler M.G. (2016). Laparoscopic sleeve gastrectomy in children and adolescents with Prader-Willi syndrome: A matched-control study. Surg. Obes. Relat. Dis..

[41] Inge T.H. (2016). A new look at weight loss surgery for children and adolescents with Prader-Willi syndrome. Surg. Obes. Relat. Dis..

[42] Polex-Wolf J., Lam B.Y., Larder R., Tadross J., Rimmington D., Bosch F., Cenzano V.J., Ayuso E., Ma M.K., Rainbow K. (2018). Hypothalamic loss of Snord116 recapitulates the hyperphagia of Prader-Willi syndrome. J. Clin. Investig..

